# Focal necrosis mimicking breast cancer following coronary bypass grafting

**DOI:** 10.1186/s12957-017-1178-4

**Published:** 2017-05-30

**Authors:** Oldřich Coufal, Tomáš Ostřížek, Petr Krsička, Eva Lžičařová, Rudolf Nenutil, Monika Procházková, Beatrix Bencsiková, Peter Grell, Roman Šefr

**Affiliations:** 1 Department of Surgical Oncology, Masaryk Memorial Cancer Institute, Zluty kopec, 7, 656 53 Brno, Czech Republic; 2 Centre of Cardiovascular and Transplantation Surgery, Pekarska 53, 656 91 Brno, Czech Republic; 3 Department of Oncological and Experimental Pathology, Masaryk Memorial Cancer Institute, Zluty kopec, 7, 656 53 Brno, Czech Republic; 4 Department of Radiology, Masaryk Memorial Cancer Institute, Zluty kopec, 7, 656 53 Brno, Czech Republic; 5 Department of Comprehensive Cancer Care, Masaryk Memorial Cancer Institute, Zluty kopec, 7, 656 53 Brno, Czech Republic; 6 Department of Surgical Oncology, Faculty of Medicine, Masaryk University, Zluty kopec, 7, 656 53 Brno, Czech Republic

**Keywords:** Coronary artery bypass, Mammary arteries, Necrosis, Breast, Carcinoma

## Abstract

**Background:**

Breast cancer can be diagnosed easily in most cases. However, occasionally, we are faced with some conditions that can mimic it. These may include inflammations, benign tumors, cysts, hematomas, or, more rarely, focal necrosis.

**Case presentation:**

This report presents a case of focal breast necrosis following myocardial revascularization with the left internal mammary artery, which is a very rare condition, with only few cases described in the literature. The necrosis becomes usually apparent a few days or weeks after the surgery and is often coincidental with the dehiscence of sternotomy with necrosis of wound edges. As it mostly affects the skin, it can be easily recognized. Also, our patient developed a dehisced sternotomy shortly after the surgery but there were no obvious objective changes on the breast. The condition was first dominated only by non-specific subjective symptom—pain. Later, a lump in the breast occurred, when the sternotomy had already healed. Moreover, an enlarged lymph node was palpable in the axilla. Because of non-typical symptoms, the condition was suggestive of breast cancer for a relatively long time. The patient had suffered from a very strong pain until she was treated by mastectomy with a good clinical result.

**Conclusions:**

Mammary necrosis following the coronary artery bypass is rare. In most cases, it manifests on the skin shortly after the surgery concurrently with dehisced sternotomy, so it can be easily diagnosed. However, in sporadic cases, the symptoms may occur later and may mimic breast cancer. Our objective is to raise awareness of this rare condition.

## Background

Breast cancer is a frequent malignancy in women. In most cases, it can be diagnosed easily; however, occasionally, clinicians are faced with some conditions that can mimic it. These may include inflammations, benign tumors, cysts, hematomas, or, more rarely, focal necrosis. Relatively common is fat necrosis following plastic surgery or trauma. By contrast, ischemic necrosis caused by using the arteria thoracica interna (internal mammary artery (IMA)) for myocardial revascularization is very rare. Under normal conditions, IMA contributes the greater proportion of blood flow to the breast [1]. However, there is an extensive network of collateral branches, and except for the period of breast-feeding, the breast itself is not very demanding in terms of perfusion. Internal mammary artery is usually considered the vessel of first choice for myocardial revascularization [2]. This article deals with the ischemic necrosis following coronary artery bypass grafting with the use of IMA which was for a relatively long time suggestive of breast cancer.

## Case presentation

A 69-year-old female patient with coronary artery disease was indicated for myocardial revascularization surgery. She had a medical history of hypertensive heart disease, hyperlipidemia, hyperuricemia, gastro-oesophageal reflux disease, and renal insufficiency with mild retention of nitrogen compounds. She was a former smoker and obese (body mass index of 39.2) with large breasts (macromastia).

Myocardial revascularization was performed as a double coronary artery bypass, using the left IMA, with no perioperative complications. Shortly after the surgery, a septicemia, mechanical instability of the sternum and mediastinitis ensued. Targeted antibiotic therapy was administered, the sternotomy wound was cut open, and vacuum-assisted closure was used to drain the wound. The sternotomy was successfully re-sutured 5 weeks after the surgery.

During the treatment of the dehisced sternotomy, the patient was complaining of pain in her left breast which persisted even after the sternotomy had healed. Ultrasonographically, there was a hyperechoic edema of the fat of 2 cm width in the central area of the breast. Although the ultrasound did not confirm any obvious focal structures, the whole clinical condition aroused suspicions of an oncological issue. Seven weeks after the surgery, oncological consultation was undertaken. The consultant clinical oncologist observed slight reddening in about one third of the breast with the maximum in the central area and inner quadrants, where a painful lump 8 × 6 cm in size was palpable. Laboratory tests showed slightly elevated serum level of C-reactive protein. With regard to the case history of wound infection, the condition was suggestive of mastitis. Empirical antibiotic therapy was recommended. During the check-up two weeks later, the antibiotic therapy had no effect and there was one new solid movable lump 2–3 cm in size in the left axilla suspicious of a pathologic lymph node. Ultrasonography showed an edema and the destruction of the normal structure of the mammary gland, without any well-defined masses or fluid collections. Thus, the significant discrepancy between the large palpable lump in the breast and the inconclusive sonographic findings still remained.

**Fig. 1:**
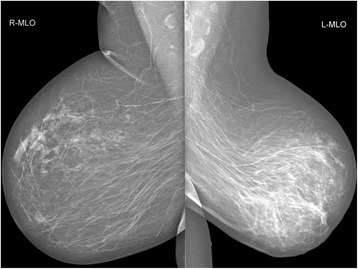
Asymmetrical mammographic findings suggestive of inflammatory carcinoma of the left breast

After another 3 weeks, ultrasound examination finally revealed a hyperechoic, poorly defined mass in the left breast with irregular hypoechoic zones and acoustic shadowing. Supported by the evidence of nipple retraction and skin thickening, the condition was now suggestive of inflammatory carcinoma. Mammographically, there were asymmetrical findings on the left breast that were smaller than the other one, with thickened areolar skin and nipple retraction. Most of the breast was edematous with the destruction of the normal structure of the gland, of higher density, which further supported the diagnosis of an inflammatory carcinoma (Fig. 1).

**Fig. 2:**
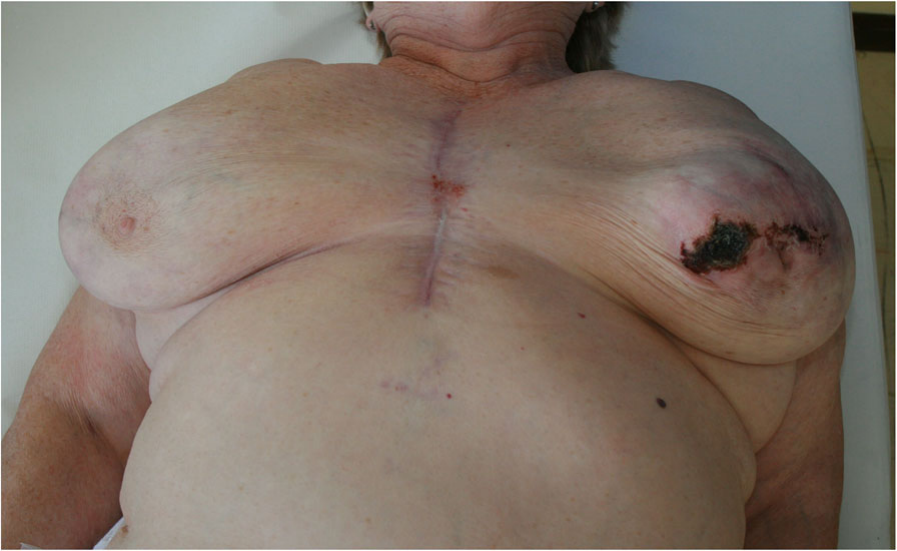
Clinical findings at the time of diagnosis

A core cut biopsy from the breast mass was undertaken with a finding of involutional breast gland with fat necrosis of unknown origin. For the first time—more than 3 months after the surgery—the suggestion was made that the condition was a result of secondary ischemic insufficiency following a harvest of the left IMA. The patient was weepy, and she was suffering from excruciating pains that prevented her from sleeping. Clinically, there was a solid palpable lump 10–15 cm in size, located in the central area of the left breast. At that time, a local cutaneous necrosis around the nipple-areola complex occurred (Fig. 2). There were solid palpable nodes in both axillae, more distinct on the left side, 3 cm in size. Given the extent of the process and the very strong pain, a total mastectomy was recommended.

**Fig. 3:**
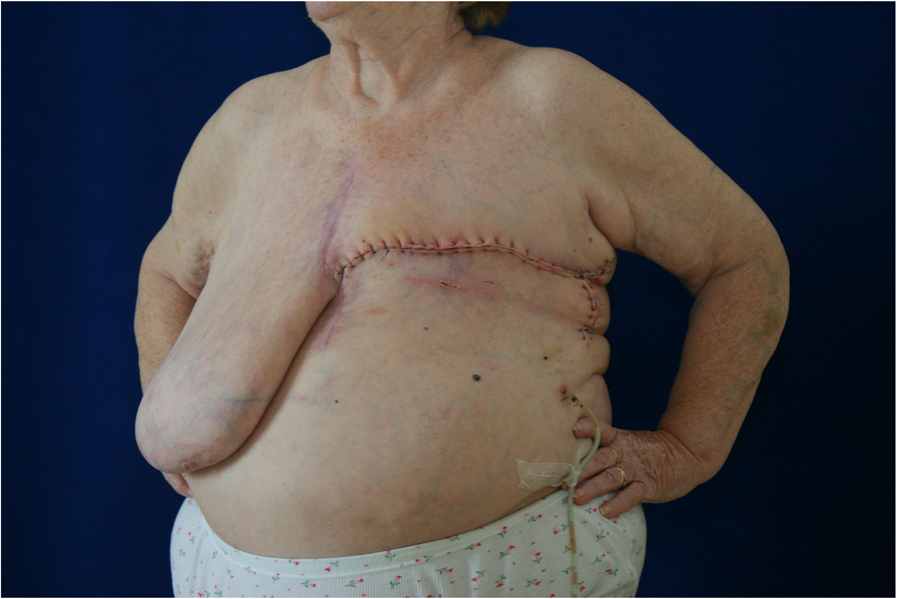
Condition after mastectomy with a fish-shaped incision

The mastectomy was carried out 19 weeks after the revascularization surgery. Because of obesity and skin folds, a lateral fish-shaped incision was used [3]. The weight of the mastectomy resection specimen was 1753 g. The wound healed by primary intention, and no complications were observed (Fig. 3). Histopathological examination of the resection specimen showed an extensive coagulative necrosis with demarcation lines and lipophagic reaction in the periphery. In the axillary tail of the breast, a lymph node of the size 25 mm with reactive changes was found. As for the vessels, no degenerative changes or calcifications were observed.

Five weeks after the mastectomy, the patient’s condition was generally better; she was optimistic and experiencing no pain. The mastectomy wound had healed up, and the patient was referred to her general practitioner with no need for future oncological check-ups.

## Discussion

Breast necrosis following IMA harvest for myocardial revascularization is a rare condition; there are only few cases described in the literature [4–13]. All of them occurred in female patients, always on the left side. Most of the patients suffered from chronic renal insufficiency, often with the need of hemodialysis, sometimes accompanied by calciphylaxis, which is the systemic medial calcification of small and medium blood vessels of the skin, leading to tissue ischemia and painful skin ulcerations [5]. In the case report of Bintoudi et al., the authors demonstrate extensive vascular calcifications on the mammogram [10], and Rashid et al. even suggest using mammography for evaluation of the rates of vascular insufficiency [5]. In other case reports, neither renal insufficiency nor calcifications are mentioned; the authors are thinking of different possible dominant risk factors, namely a macromastia, since all patients were women, mostly with large breasts. An anomalous vascular supply to the breast, mainly from the IMA, is also mentioned [6, 11]. In the three cases described by Wong et al., the construction of the pectoralis major myocutaneous flap, which was used for surgical treatment of dehisced sternotomy, may had contributed to the breast necrosis [9]. In another case, described by Rosato, blood perfusion of the mammary gland was damaged by the previous treatment of breast cancer, which included partial mastectomy, axillary dissection, and radiotherapy [7]. The most patients with breast necrosis following IMA harvest also suffered from diabetes mellitus and hypertension. It is most likely that this rare condition is caused by a combination of the various aforementioned risk factors.

The necrosis of the breast tissue becomes usually apparent a few days or weeks after the revascularization cardiac surgery and is often coincidental with the dehiscence of sternotomy with necrosis of wound edges. As it mostly affects the skin, it can be easily recognized. Also, our patient developed a dehisced sternotomy shortly after the surgery, but at first, there were no obvious objective changes in the breast region. The affection of the breast was first dominated only by non-specific subjective symptom—pain. Only later, when the sternotomy has healed, an irregular lump appeared, accompanied by signs typical of breast cancer—erythema, nipple retraction, and enlarged axillary lymph node. At that time, there were no obvious signs of skin necrosis. Not event mammography helped to distinguish fat necrosis from carcinoma. There were no typical signs of necrosis with calcifiactions and oil pseudocysts (eggshell calcifications). There was just one small oil pseudocyst, which probably was not linked to the whole condition. The structure of the breast was generally condensed and blurred with the thickening of the skin in the central part of the breast and with the nipple retraction. The findings thus looked like an inflammation or an inflammatory carcinoma (Fig. 1). Given the non-specific symptoms, our patient was diagnosed with ischemic necrosis as late as 14 weeks (3 months) after the revascularization procedure. Such a mimicking of mammary carcinoma has been described in only two case reports where a biopsy had to be used for a differential diagnosis [5, 10].

According to published works, the extent of necrosis may range from small focal lesions to large necrotic areas spreading across the breast or to the front abdominal wall in the hypochondriac regions [6, 11]. As for the treatment, debridement, antibiotic therapy, and healing by conservative ways through granulation were sufficient in the two of the so far described cases [4, 8]. In other cases, the condition was handled by more extensive resections, through partial or total mastectomy [5, 7, 10, 11, 13], sometimes with resection of thoracic or abdominal walls [5, 7, 10]. Subsequent surgical reconstructions with skin grafts or more complex fasciocutaneous or musculocutaneous flaps occasionally had to be used [5, 6, 9, 11]. In several cases, healing was complicated and required repeated surgeries [6, 9, 11]. Two patients even died due to the postoperative complications [9, 12].

In our case, the dehisced sternotomy was handled conservatively, i.e., by vacuum-assisted closure and resuture, with complete healing occurring within 5 weeks from the original surgery. As for the later manifested mammary necrosis, it was also possible to consider a conservative approach, as in the works of Har-Shai and Ricci. From an oncological point of view, there was no need for resection, since malignancy was ruled out by the biopsy. Nevertheless, due to severe pain both patient and attending physician supported a more rapid solution. Given the extent of the process, a total mastectomy was opted for, and resected mass involved all affected tissue including the necrotic skin. There was no need for any further plastic surgical reconstructions, as opposed to the works of and Rashid et al. and Morris et al. [5, 13] (post-mastectomy skin-graft), or Harish et al. [11] (vertical rectus abdominis myocutaneous flap). Post-mastectomy healing is undoubtedly influenced by the extent of necrosis—if necrotic tissue is completely included in the resected mass, healing by first intention may be expected, as what occurred in our case and some other published works [7, 10].

The surgery had an immediate analgesic effect, our patient experienced great relief. So far, published works do not discuss the issue of pain too much. It is mentioned only by Rosato, with necrosis being solved, as in our case, by total mastectomy with healing by first intention and analgesic effect.

## Conclusions

Mammary necrosis following the coronary artery bypass is a rare condition. In most cases, it manifests on the skin shortly after the surgery concurrently with dehisced sternotomy, so it can be easily diagnosed. However, in sporadic cases the symptoms may occur later and may not be characteristic. The condition can mimic a breast cancer as was demonstrated by our case. The treatment of mammary necrosis is surgical, and the radicality of the procedure depends on the extent of necrosis. In our case, a total mastectomy was opted for, which allowed healing by first intention and had a positive and rapid analgesic effect. An earlier diagnosis would not necessarily have changed the extent of the surgical intervention, but at least it could have spared the patient a few days or weeks of pain and stress.

## Abbreviation

IMA: Internal mammary artery
